# The DAIR (debridement, antibiotics and implant retention) procedure for infected total knee replacement – a literature review

**DOI:** 10.1051/sicotj/2016038

**Published:** 2017-01-11

**Authors:** Sultan Naseer Qasim, Andrew Swann, Robert Ashford

**Affiliations:** 1 Orthopaedic Resident, Leicester Orthopaedics, University Hospitals of Leicester Leicester LE1 5WW UK; 2 Consultant Microbiologist, University Hospitals of Leicester Leicester LE1 5WW UK; 3 Consultant Orthopaedic and Sarcoma Surgeon, Leicester Orthopaedics, University Hospitals of Leicester Leicester LE1 5WW UK

**Keywords:** Debridement and implant retention, DAIR, Infected total knee arthroplasty, Prosthetic joint infection

## Abstract

Prosthetic joint infection (PJI) is a devastating complication in total knee arthroplasty (TKA) and third most common cause of revision of TKA with significant morbidity and surgical challenges. Treatment options include non-operative measures with long term antibiotic suppression, debridement and implant retention (DAIR), one- or two-stage revision arthroplasty, arthrodesis and amputation. Implant retention without infection is ideal and DAIR has been reported to have variable success rates depending on patient factors, duration of infection, infecting micro-organisms, choice of procedure, single or multiple debridement procedures, arthroscopic or open, antibiotic choice and duration of antibiotic use. We present a thorough literature review of DAIR for infected TKA. The important factors contributing to failure are presence of sinus, immunocompromised patient, delay between onset of infection and debridement procedure, Staphylococcal infection in particular Meticillin Resistant *Staphylococcal aureus*, multiple debridement procedures, retention of exchangeable components and short antibiotic duration. In conclusion DAIR can be successful procedure to eradicate infection in TKA in selective patients with factors contributing to failure taken into account.

## Introduction

Knee replacement is an effective procedure for end-stage knee arthritis. According to the National Joint Registry report of 2013 [1], 22% of revision procedures were performed for infection and according to the National Joint Registry report of 2014 [1] infection is the third most common cause of revision after aseptic loosening and pain with 1.06 revision per 1000 patient-years. It is, however, one of the most dreaded complications for orthopaedic surgeons because of significant morbidity and because of the surgical challenges it poses.

The treatment options for an infected total knee arthroplasty (TKA) are usually surgical as antibiotics in isolation are not an appropriate treatment unless there is significant co-morbidity providing a relative contra-indication for surgery [2]. The surgical options include washout, debridement, antibiotics and implant retention (DAIR), one- or two-stage revision total knee arthroplasty, arthrodesis and amputation. Prosthetic joint infection (PJI) surgery also poses significant cost implications [3] as it could require multiple procedures, prolonged antibiotics, lengthy hospital stays and more expensive implants for revision surgery.

Two-stage revision arthroplasty is considered the gold standard for an infected prosthesis. However, it poses significant challenges to both patient and surgeon. Two operations with a substantial period of reduced mobility and significant anaesthetic and surgical risks are major concerns for the patient. There are significant challenges for the surgeon in removing a well-fixed prosthesis with removal of bone cement. This poses risk of significant damage to the remaining bone stock, making reconstruction difficult with increased risk of perioperative and postoperative complications and potentially compromising the soft tissue envelope.

Implant retention without infection is the ideal end result of treatment for an infected total knee arthroplasty. Some surgeons prefer to perform debridement of the knee, particularly in the case of acute infection, to reduce the infective organism load and supplement debridement with systemic antibiotics. Their aim is to retain the implant and to avoid further more invasive/complex surgery. There are no randomized controlled or prospective trials. The literature consists of various retrospective series both in Orthopaedic and Microbiology journals with variable opinions over each aspect of DAIR.

We, therefore, present a thorough review of literature for role of the DAIR procedure as a treatment option for infected TKA.

## Materials and methods

MEDLINE was searched using the PubMed and Embase interface to identify relevant studies pertaining to debridement and implant retention in infected knee arthroplasty up to June 2015. Keyword searches used were “prosthetic joint infection”, “infected knee arthroplasty”, “infected knee replacement”, “debridement”, “implant retention” and “DAIR”. Inclusion criteria included all articles which described implant retention for infected knee arthroplasty published in the English language or with abstracts in English.

**Figure 1.**
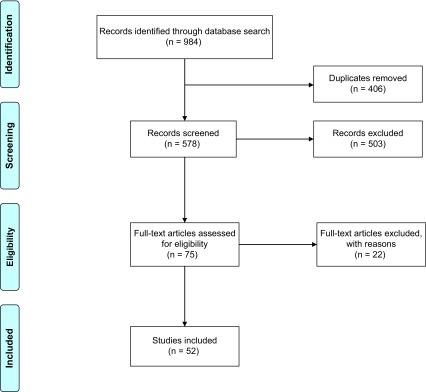
PRISMA flowchart.

PRISMA guidelines were used as shown in Figure 1.

Data extracted from the studies included patient factors, duration of symptoms, micro-organisms identified, procedure performed – open/arthroscopic and single/multiple debridement, duration of antibiotic and long-term results.

The principal outcome measure was failure of the procedure and was defined as failure to eradicate infection and need for further procedure that included revision, arthrodesis or amputation. Some of the key studies are shown in Table 1.

**Table 1.** Key studies.

Study (year of publication)	Number of patients	Success rate	Comments
Segawa et al. [4] (1999)	81	85%	Major factor associated with treatment failure was compromised immune status. Bone loss and necrosis of soft tissues also contributed.
Marculescu et al. [5] (2006)	99	60%	Sinus tract and duration of symptoms >8 days independent risk factors for failure.
Hsieh et al. [30] (2009)	346	GN PJI 27%	Debridement alone has a high failure rate and should not be attempted when the duration of symptoms is long.
		GP PJI 47%	
Deirmengian et al. [23] (2003)	31	35%	Success rate of DAIR depends on involved pathogen. Streptococcal or *Staphylococcus epidermidis* has better success rate if done within 2–3 days of symptom onset.
Triantafyllopoulos et al. [29] (2014)	78	55%	MRSA success rate of 45.1%.
Zürcher-Pfund et al. [32] (2013)	21	33%	0/3 for MRSA.
Geurts et al. [13] (2013)	20	85%	Rate of failure associated with number of debridement procedures.
Gardner et al. [12] (2011)	44	43%	*Staph aureus* infection with greater failure.
Choi et al. [34] (2011)	32	31%	Staphylococcal infection and polyethylene non-exchange major factors for failure.
Koyonos et al. [33] (2011)	138	31% Acute	Staphylococcal infection, most significant indicator of failure.
		44% Acute delayed	
		28% Chronic	
Azzam et al. [22] (2010)	104	44%	DAIR has a low success rate. Effective for low virulence organisms.
Vilchez et al. [21] (2011)	65	Haematogenous PJI – 45%	Haematogenous PJI due to *S. aureus*, using debridement with implant retention, had a worse outcome than early post-surgical infections.
		Early PJI – 75%	
Bradbury et al. [26] (2009)	19	18%	The total success rate of open irrigation and debridement with component retention (ODCR) in acute periprosthetic MRSA knee infection was 18%.
Chung et al. [45] (2014)	16	100%	62.5% Arthroscopy alone; the rest needed further open debridement.
Liu et al. [43] (2013)	17	88%	Arthroscopic debridement with continuous irrigation and suction effective in prosthesis retention.
Mont et al. [44] (1997)	24	80%	DAIR effective for early PJI.
Trebse et al. [7] (2005)	24	86%	DAIR effective for treating early PJI.
Pavoni et al. [2] (2004)	34	91%	Treatment with long-term antibiotics alone in PJI can yield good results.
Sherrell et al. [52] (2011)	83	66%	High failure rate after two-stage revision is worse in patients previously treated with DAIR.
Lora-Tamayo et al. [28] (2013)	345	55%	The use of rifampicin may have contributed to homogenizing MSSA and MRSA prognoses, although the specific rifampicin combinations may have had different efficacies.
Puhto et al. [49] (2012)	86	89.5%	Shorter course of antibiotics is as effective as longer antibiotics course.
Cobo et al. [25] (2011)	117	57.3%	DAIR recommended in early PJI
Byren et al. [48] (2009)	112	72%	DAIR effective in PJI

## Results and discussions

### Patient factors

A patient’s innate immunity is a well-established factor for infection control after prosthetic joint infection (PJI). The risk factors for failure after DAIR are no different to any other surgical procedure. The condition of local soft tissues is of particular significance [4]. Persistence of a sinus has been shown to be a risk factor for failure [5–7] and a contraindication to DAIR. Furthermore, DAIR relies on patient immunity to combat infection [4, 6, 8]; hence, it is not a suitable procedure in the immunocompromised individual. It may be appropriate in individuals with significant morbidity who are unsuitable for alternative surgical options with higher morbidity.

### Duration of symptoms

Various definitions of PJI have been described. The American Academy of Orthopaedic Surgeons working party proposed diagnostic criteria for PJI [9] to aid decision making. Cui et al. [10] classified infections into four types as shown in Table 2.

**Table 2.** Types of prosthetic joint infection according to Cui et al. [10].

Acute postoperative	≤4 weeks postoperative
Late chronic	Indolent infection >4 weeks postoperative
Acute haematogenous	Acute onset at the site of a previously well-functioning prosthetic joint
Positive intra-operative culture	Clinically unapparent infection with two or more positive intra-operative cultures

Success rates of 28%–62% have been shown with DAIR for late chronic or established Prosthetic Joint Infection as compared to 31%–100% with acute infections. Furthermore, acute postoperative infection has shown better results as compared to haematogenous spread [6, 11–21].

### Microbiology

Many organisms have been isolated in infected total knee arthroplasty. Common pathogens include meticillin-sensitive and meticillin-resistant *Staphylococcus aureus* (MSSA and MRSA), coagulase-negative staphylococci, streptococci and Gram-negative bacteria. *Staphylococcus aureus* is generally pyogenic and often accounts for acute postoperative infections. Coagulase-negative staphylococci are often associated with late chronic or clinically unapparent infections, typically as a result of biofilm production.

Multiple studies have shown *Staphylococcus aureus* infection to be an independent risk factor for failure to eradicate infection [12, 18, 22–25]. MRSA, in particular, has shown high failure rates with DAIR [26–29]. Gram-negative organisms have shown a variable outcome in failure rates as compared to Gram-positive organisms [21, 30, 31]. The decision to retain implant should depend on pathogen e.g. not to retain in cases of MRSA infection as echoed by Zürcher-Pfundet et al. [32]. DAIR has shown to be particularly effective in patients who are not immunocompromised and with PJIs caused by a low virulence organism e.g. coagulase-negative staphylococci [12, 23, 33].

## Procedure

The principles of debridement for infected arthroplasty are to withhold antibiotics and to aspirate the joint to identify organism prior to surgery. The surgical procedure includes removal of skin margins, excision of any sinuses, radical synovectomy and exchange of removable implants (polyethylene insert in case of total knee arthroplasty). In the case of a modular total knee replacement, removal of the polyethylene tibial insert is very important in order to gain access to posterior aspect of the joint. Choi et al. [34] showed that not exchanging the polyethylene insert was an independent risk factor for failure. Based on this, patients with an all-polyethylene tibial component might not be good candidates for implant retention.

The joint must have a thorough lavage. Recently there has been increased interest in using chlorhexidine gluconate 0.05%. Schwechter [35] undertook an *in vitro* research to investigate the replication of adherence of MRSA to orthopaedic implants by creating a biofilm model using titanium discs. They found chlorhexidine gluconate to be most effective in reducing bacterial colony counts and was superior to pulsed saline lavage.

A suction drain should be left in situ until there is minimal output. If drainage persists or if the infection fails to settle then consideration has to be given to a further debridement procedure. Continuous closed irrigation has not proven to be any more effective than standard procedure with primary closure and in situ drain [36].

Martel-Laferrière et al. [37] and others [22, 38, 39] have proposed treatment algorithms but Gardener highlights that no single treatment algorithm is preferable to any other [28].

### Arthroscopic versus open

A DAIR procedure cannot be carried out arthroscopically because this does not allow adequate debridement or exchange of the polyethylene insert. Some authors [40–42] have utilized arthroscopic washout in acute infections. Ilahi et al. [41] report 100% success in seven patients and Liu et al. [43] report 88% success rate in 15 patients they treated but others report a much lower success rate [42, 45] when compared to open debridement. We do not advocate this as the procedure of choice.

### Single versus multiple

Most studies would regard the need for a further procedure as failure of the index procedure. Vilchez et al. [39] showed that repeat procedure was an independent predictor of failure. Therefore, repeated DAIR procedures are not recommended and indicate failure and need for alternative procedure [23, 37]. However, Mont et al. [44] performed that repeated DAIR is successful in early infection.

### Antibiotic choice

The choice of antibiotic will depend on a number of factors including susceptibilities of pathogens isolated, route of administration and likely duration of treatment. Regimens for intravenous or highly bioavailable oral antimicrobial treatment of particular pathogens have been recommended [45]. Individual regimens should be chosen following discussion between orthopaedic surgeons and microbiologists or infectious disease specialists.

### Antibiotic duration

This by far is the most controversial issue in the treatment of prosthetic joint infection. The duration varies from no extended regimen [47] to indefinite [2]. Byren et al. [48] treated their patients with systemic antibiotics for an average of 1.5 years and cited antibiotic duration as a major risk factor for failure. Puhto et al. [49] found no difference in the recurrence of infection when antibiotics were used for three or six months post-procedure, thus advocating shorter duration. Laffer et al. [16] showed similar results. Bernard et al. [50] showed that even six weeks of postoperative antibiotics had similar results as compared to long-term use. Moran et al. [51] recommend the use of broad spectrum agent in the early empirical antibiotic regime for PJI. We believe, however, that DAIR may require longer antibiotic use than in revision procedure. Nevertheless, it is agreed that the duration depends on the virulence of the offending pathogen, the need for repeat procedures and host factors. In some cases, chronic suppressive antibiotic therapy may be appropriate, especially in those for whom further surgical options are not possible for either medical or surgical reasons.

### Long-term results

Failure of a DAIR procedure will necessitate alternative surgical options with two-stage procedure being the gold standard. Gardner et al. [12], Choi et al. [34] and Sherrell et al. [52], however, showed that a two-stage procedure, in a patient with previous debridement, has an inferior success rate and outcome when compared to a primary procedure. In the study by Choi [34], in 22 failed initial DAIR procedures, staged revision (11 knees), repeated incision and drainage (I&D) (11 knees), fusion (two knees), amputation (two knees) and resection (one knee) were performed.

### Recommendation

We recommend a DAIR procedure in the acute postoperative period within four weeks of surgery or acute haematogenous infection of TKA within two weeks of onset. The procedure should be open rather than arthroscopic and the polyethylene insert exchanged where possible. Postoperative antibiotics should be given for six weeks and the progress monitored by clinical examination and inflammatory markers. Immunocompromise, MRSA infection, poor condition of local soft tissues and failure of one DAIR procedure should prompt revision arthroplasty.

## Conclusion

Debridement, antibiotics and implant retention (the DAIR) procedure can be a successful treatment option for PJI in TKA. It can effectively eradicate infection, resulting in improved functional outcome and a reduction in the need of more extensive surgery which may be associated with far greater morbidity. Individual factors affecting outcome include patient factors, presence of sinus, duration of infection, virulence of organism, meticulous procedure, exchange of polyethylene insert and duration of appropriate antibiotic use. Failure of a DAIR procedure generally requires more radical procedures such as revision arthroplasty.

## Conflict of interest

Authors SQ, AS, RA certify that they have no financial conflict of interest (e.g. consultancies, stock ownership, equity interest, patent/licensing arrangements, etc.) in connection with this article. The institution of authors SQ, AS, RA (University Hospitals of Leicester NHS Trust) has not received any funding related to this article.
